# Prevalence, antibiotic resistance patterns, and biofilm formation ability of *Enterobacterales* recovered from food of animal origin in Egypt

**DOI:** 10.14202/vetworld.2023.403-413

**Published:** 2023-02-28

**Authors:** Shimaa N. Edris, Ahmed Hamad, Dina A. B. Awad, Islam I. Sabeq

**Affiliations:** Department of Food Hygiene and Control, Faculty of Veterinary Medicine, Benha University, Benha 13736, Egypt

**Keywords:** biofilm, *Enterobacteriaceae*, food safety, multidrug resistance

## Abstract

**Background and Aim:**

The majority of animal-derived food safety studies have focused on foodborne zoonotic agents; however, members of the opportunistic *Enterobacteriaceae* (Ops) family are increasingly implicated in foodborne and public health crises due to their robust evolution of acquiring antimicrobial resistance and biofilms, consequently require thorough characterization, particularly in the Egyptian food sector. Therefore, this study aimed to determine the distribution and prevalence of *Enterobacteriaceae* family members in animal-derived foods, as well as their resistance to important antimicrobials and biofilm-forming potential.

**Materials and Methods:**

A total of 274 beef, rabbit meat, chicken meat, egg, butter, and milk samples were investigated for the presence of *Enterobacteriaceae*. All isolated strains were first recognized using traditional microbiological techniques. Following that, matrix-assisted laser desorption ionization-time of flight mass spectrometry was used to validate the *Enterobacteriaceae’s* identity. The isolated enterobacteria strains were tested on disk diffusion and crystal violet quantitative microtiter plates to determine their antibiotic resistance and capacity to form biofilms.

**Results:**

There have been thirty isolates of *Enterobacteriaceae* from seven different species and four genera. Out of the three food types, *Pseudomonas aeruginosa* had the highest prevalence rate (4.1%). With three species, *Enterobacter* genera had the second-highest prevalence (3.28%) across five different food categories. In four different food types, the *Klebsiella* genera had the second-highest distribution and third-highest incidence (2.55%). Almost all isolates, except three *Proteus* *mirabilis*, showed prominent levels of resistance, particularly to beta-lactam antibiotics. Except for two *Enterobacter* *cloacae* and three *P. mirabilis* isolates, all isolates were classified as multidrug-resistant (MDR) orextensively multidrug-resistant (XDR). The multiple antibiotic resistance index (MARI) of the majority of isolates dropped between 0.273 and 0.727. The highest MARI was conferred by *Klebsiella* *pneumoniae*, at 0.727. Overall, 83.33% of the isolates had strong biofilm capacity, while only 16.67% exhibited moderate capacity.

**Conclusion:**

The MDR, XDR, and strong biofilm indicators confirmed in 83.33% of the currently tested *Enterobacteriaceae* from animal-derived foods suggest that, if not addressed, there may be rising risks to Egypt’s economy and public health.

## Introduction

Animal-derived foods are an important source of nutrition for many people worldwide, but their consumption may pose several health risks. This places the safety of animal-derived foods at the forefront of societal concerns and is one of the current and ongoing challenges for food producers [1, 2]. One of these biological foodborne health risks is *Enterobacteriaceae*. The *Enterobacteriaceae* is a sizable, diverse family of Gram-negative rods that includes bacteria that naturally live in the gastrointestinal tracts of mammals and can also exist and proliferate in other environments. *Enterobacteriaceae* not only contribute to food spoilage, but they also pose a microbiological risk to consumers. Consumption of raw or undercooked meat and cross-contaminated food products increases human infection among such family members [3]. Human wound infections, urinary tract infections (UTIs), gastroenteritis, meningitis, pneumonia, septicemia, and hemolytic uremic syndrome can all be caused by *Enterobacteriaceae*. *Enterobacteriaceae* have always been required as indicator bacteria for the microbiological quality of food and the hygiene level of manufacturing processes due to these risks [4, 5].

Another risk that has become a significant global health concern in the 21^st^ century and poses a growing threat to both human and animal health is antimicrobial resistance (AMR). The critical cause is the indiscriminate use of antibiotics in animal husbandry [6, 7]. Unfortunately, *Enterobacteriaceae* contamination in food is not only associated with spoilage and illness, but the most serious issue is their ability to rapidly acquire and transfer resistance (mobile gene), particularly multidrug-resistant (MDR), to other pathogens, including *Escherichia coli* and *Salmonella* [8]. Most Gram-negative bacteria (GNB), including *Enterobacteriaceae*, have long been blamed for being the most common MDR carriers [9]. Most studies have focused on the AMR of zoonotic *Enterobacteriaceae* members in animal-derived foods [10–12].

Biofilm is another problematic food safety issue for food producers, with multiple causes, including bacteria as the primary contributor and profound consequences on food processing efficiency. Biofilm in food-related environments and equipment is composed of either homogeneous or heterogeneous microbial communities living in a self-secreted matrix of extracellular polymeric substances [13, 14]. Bacterial biofilms play a crucial role in AMR because they are more resistant to antimicrobial agents than planktonic cells [15]. In addition, the ability of microorganisms to form biofilms, which differ greatly between bacterial populations, is one of the factors that determine their virulence and ability to survive in unfavorable environments like preservation. Therefore, the prevalence and associated potential risk level of biofilm-forming *Enterobacteriaceae* was a focus of earlier studies [16, 17].

The presence of *Enterobacteriaceae* in large quantities in manure, soil, irrigation water, or animal feces raises the risk of contamination of animal-derived commodities. Furthermore, *Enterobacteriaceae* coexisting and sharing mobile resistance genes as well as biofilm formation with other pathogens in animal-derived foods such as meat, poultry, milk, and eggs may pose more serious risks such as MDR bacteria, classifying these foods as potential MDR vectors [18–20]. The current screening study was conducted in response to the increased potential threat posed by *Enterobacteriaceae* strains, with the goals of determining the distribution and prevalence of *Enterobacteriaceae* family members in animal-derived foods, as well as their resistance to critical antimicrobials and biofilm-forming capacity.

## Materials and Methods

### Ethical approval

The study was approved by the Care and Use Committee Research Ethics, Faculty of Veterinary Medicine, Benha University (BUFVTM, 01/01/23), Egypt.

### Study period and location

The study was conducted from September 2019 to June 2021 in Al Qalyubia Governorate, Egypt.

### Sample collection

A total of 274 animal-derived foods were collected and microbiologically tested for the presence of *Enterobacteriaceae*. Animal-derived foods included beef, chickens, rabbits, milk, butter, and eggs. The food product samples were collected from supermarkets, local markets, and retail shops. Samples were collected and transported to the laboratory in sterile plastic bags within ice boxes within 1 h for microbiological analysis.

### Isolation and identification of *Enterobacteriaceae* strains

A standard cultivation method recommended by ISO [21] was used with some modifications for the isolation and identification of *Enterobacteriaceae*. In brief, 25 g of each food sample was weighed and homogenized in a sterile stomacher bag (Stomacher 400R, Seward, UK) with 225 mL of buffered peptone water for 2 min at 10× *g* followed by overnight incubation at 37°C in a sterile stomacher bag (Stomacher 400R) for peri-enrichment. The pre-enrichment broth was then transferred to 10 mL of *Enterobacteriaceae* enrichment broth (Oxoid, UK) and incubated at 37°C for 24 h. To isolate *Enterobacteriaceae* species, each suspected tube from the selectively enriched medium was streaked onto violet red bile glucose agar plates (BioLife, USA) and incubated at 37°C for 18–24 h. Suspect colonies with typical *Enterobacteriaceae* morphology were biochemically confirmed using the API 20E® system (BioMerieux, France).

### Identification by matrix-assisted laser desorption ionization-time of flight mass spectrometry (MALDI-TOF MS)

Presumptive *Enterobacteriaceae* isolates were confirmed using MALDI-TOF MS (VITEK^®^ MS, database version 3, BioMerieux). As calibration and internal identification control, *E. coli* ATCC 8739 strain cells were inoculated on the calibration spots. The results were interpreted following the manufacturer’s recommendations. The peaks from the spectrum were compared to the typical spectrum for a species, genus, or family of microorganisms, thus, resulting in isolate identification.

### Antimicrobial susceptibility testing via disk diffusion method

The *Enterobacteriaceae* isolates were subcultured twice on tryptic soy agar plates at 37°C for 20 h to prepare a bacterial suspension. The antimicrobial susceptibility tests were then performed using the disk diffusion method. Four to 5 colonies from each identified *Enterobacteriaceae* species were selected from a pure culture plate. All results were interpreted following the Clinical and Laboratory Standards Institute (CLSI) [22]. In antimicrobial susceptibility determination, *Staphylococcus aureus* ATCC 25923 and *Escherichia coli* ATCC 25922 were quality control organisms. All identified *Enterobacteriaceae* species were initially tested for resistance to eleven widely available therapies in Egyptian veterinary and medical sectors, including amoxicillin-clavulanic acid (AMC, 30 μg), cefuroxime (CXM, 30 μg), ceftriaxone (CTR, 30 μg), ciprofloxacin (CIP, 5 μg), norfloxacin (NX, 10 μg), gentamicin (GEN, 10 μg), tetracycline (TE, 30 μg), trimethoprim (TR, 5 μg), vancomycin (VA, 30 μg), clarithromycin (CL, 15 μg), and erythromycin (E, 15 μg) (HiMedia, India). *Enterobacteriaceae* isolates that exhibited resistance to at least three classes of antimicrobial agents tested were considered MDR, and bacterial isolates that remained susceptible to only one or two categories were considered extensively multidrug-resistant (XDR). The multiple antibiotic resistance index (MARI) was computed by dividing the number of antibiotics to which the bacterial isolate was resistant by the total number of antibiotics used in the study [23] using the following formula:


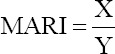


Where “X” is the number of antimicrobial agents to which bacteria revealed resistance, while “Y” is the total number of antimicrobial agents tested.

### Biofilm formation using crystal violet (CV) quantitative microtiter plate method

Each *Enterobacteriaceae* isolate was cultured overnight at 37°C in trypticase soy broth (TSB; HiMedia). Following that, 2 μL of cell suspension was injected into sterile 96-well polystyrene microtiter plates containing 198 μL of TSB. Each test comprised negative control wells containing 100 μL of uninoculated TSB. The cells were incubated for 24 h at 37°C. The wells were gently washed 3 times with 200 μL phosphate-buffered saline. The wells were dried upside down. The biofilm mass was stained with 125 μL of 0.1% CV (Oxoid). The wells were gently washed with 200 μL of distilled water 3 times and dried in inverted positions. Finally, the wells were dissolved in 200 μL of 30% acetic acid to solubilize the stain. A microplate reader (Tecan Sunrise, Jencons, UK) measured biofilm mass optical density (OD) at 595 nm. The OD cut-off (ODc) was defined as three standard deviations above the mean OD of the negative control. All the isolates were classified based on the adherence capabilities into the following categories: non-biofilm producers (OD ≤ ODc), weak biofilm producers (ODc< OD ≤ 2× ODc), moderate biofilm producers (2ODc < OD ≤ 4× ODc), and strong biofilm producers (4× ODc < OD) [24, 25].

### Statistical analysis

Statistical analysis was performed using Statistical Package for the Social Sciences (SPSS) version 20.0 (IBM Corp., NY, USA). The collected data from various food samples, antimicrobial susceptibility, and Biofilm formation results were computed using descriptive statistics such as frequency, percentage, and/or proportion.

## Results

The incidence of isolated *Enterobacteriaceae* from different food samples is presented in Table-1. Thirty enterobacteria strains from four genera and seven different species have been identified. *Pseudomonas* *aeruginosa* had the highest rate of occurrence (4.1%, 11/274), but it was determined only in three food types: Rabbit meat, butter, and milk. While *Enterobacter* genera with three species, *Enterobacter* *cloacae* (7/274), *Enterobacter* *hormaechei* (1/274), and *Enterobacter* *kobei* (1/274), had the second highest incidence (3.28%, 9/274), they also had the greatest distribution isolated from five distinct types of animal-derived food. The *Klebsiella* genera, including *Klebsiella* *pneumoniae* (5/274) and *Klebsiella* *oxytoca* (2/274), had the third-highest incidence (2.55, 7/274) and second-highest distribution in four food types. The prevalence and distribution of *Proteus* *mirabilis* were the lowest (1.094%, 11/274).

**Table-1:** Incidence of isolated *Enterobacteriaceae* from different food samples.

Strains	Food samples					

	Beef	Chicken meat	Rabbit meat	Butter	Milk	Egg
*Klebsiella pneumoniae*	—	3.03% (2/66)	1.56% (1/64)	—	1.72% (1/58)	3.70% (1/27)
*Klebsiella oxytoca*	—	—	3.13% (2/64)	—	—	—
*Pseudomonas aeruginosa*	—	—	12.5% (8/64)	28.57% (2/7)	1.72 (1/58)	—
*Proteus mirabilis*	—	1.51 (1/66)	—	—	3.45 (2/58)	—
*Enterobacter cloacae*	5.77% (3/52)	1.51% (1/66)	1.56% (1/64)	—	1.72 (1/58)	3.70% (1/27)
*Enterobacter hormaechei*	—	—	—	—	—	3.70% (1/27)
*Enterobacter kobei*	—	—	—	—	—	3.70% (1/27)

**Figure-1:**
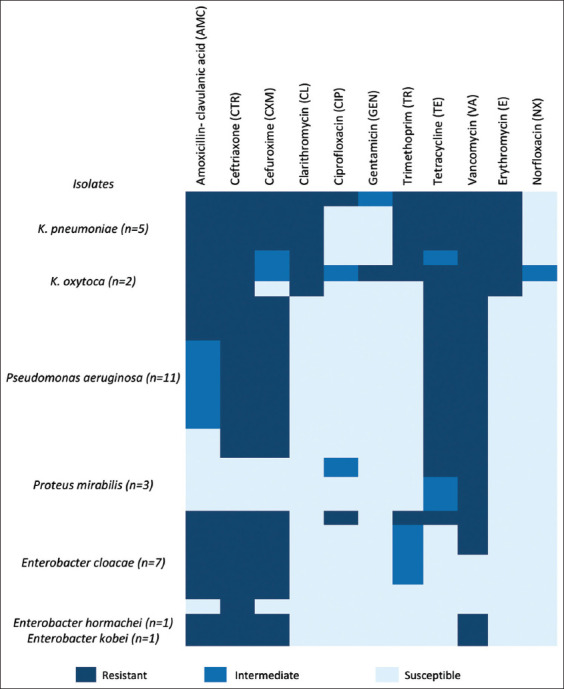
Heat map showing antimicrobial susceptibility profile of 30 *Enterobacteriaceae* isolates. Each row represents one isolate tested for susceptibility. Antimicrobial resistance was assessed by disk diffusion and cut-offs defined by CLSI guidelines [22].

The results of phenotypic antimicrobial susceptibility testing of isolated *Enterobacteriaceae* species to 11 different antibiotics are shown in Figure-1 [22]. To summarize, the majority of *Enterobacteriaceae* isolates, including *K*. *pneumoniae*, *K*. *oxytoca*, *P. aeruginosa*, *E. cloacae*, *E. hormaechei*, and *E. kobei*, demonstrated high rates of resistance, which in most isolates reached 100%, to tested beta-lactam antibiotics, including AMC, second CXM, and third-generation cephalosporins (3GCs) CXM. In detail, 100% of the five *K*. *pneumoniae* isolates were resistant to AMC, CTR, VA, TR, CL, and E, while 80% were resistant to CXM and TE, but only one isolate survived CIP. Furthermore, both *K*. *oxytoca* isolates were resistant to AMC, CTR, TE, VA, CL, and E, but only one of them was resistant to GEN and TR. Furthermore, all eleven *P. aeruginosa* isolates were resistant to CXM, CTR, TE, and VA, but only three were resistant to AMC. Except for VA resistance, all three *P. mirabilis* were susceptible to all antibiotics. All seven *E. cloacae* isolates were tested resistant to AMC, CTR, and CXM, while four screened resistant to VA, but only one isolate was resistant to CIP, TE, and TR. Finally, both *E. hormaechei* and *E. kobei* were completely resistant to AMC, CTR, CXM, and VA.

**Figure-2:**
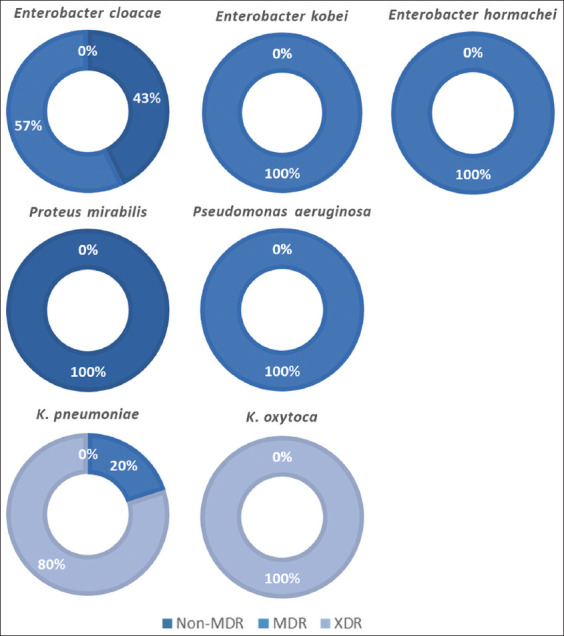
Prevalence of antimicrobial susceptibility patterns of *Enterobacteriaceae* recovered from food samples.

The antimicrobial susceptibility patterns of thirty *Enterobacteriaceae* isolates recovered from food samples are depicted in Figure-2. The current research identified three distinct antimicrobial susceptibility patterns: non-MDR, MDR, and XDR. All three *P. mirabilis* and three *E. cloacae* isolates were resistant to either one or two antibiotic classes (non-MDR), VA, and beta-lactamase inhibitors. On the other hand, all 11 *P. aeruginosa* isolates, three *E. cloacae*, and both *E. hormaechei* and *E. kobei* were classified as MDR to three antibiotic classes. Furthermore, all five *K*. *pneumoniae*, two *K*. *oxytoca*, and one *E. cloacae* are distributed as XDR to at least five antibiotic classes.

Table-2 details the antibiotic-resistant groupings detected in this study using multiple antibiotic resistance (MAR) indices. Eleven distinct AMR combinations involving single or multiple antibiotics were observed among the thirty *Enterobacteriaceae* isolates tested. Surprisingly, the MAR indices of all isolates ranged from 0.273 to 0.727, except three *P. mirabilis* isolates and one *E. cloacae* isolate, which were both 0.091. In addition, *K*. *pneumoniae* confers resistance to eight different antibiotics, reflecting the highest MARI of 0.727.

**Table-2:** Antibiotic resistance phenotypes and MARI of non-target bacterial strains isolated from different foods.

Resistance pattern	Resistance profile	Number of isolates	Number of antibiotics	MARI
*Klebsiella pneumoniae*				
I	AMC, CXM, CTR, TE, TR, VA, CL, E	3	8	0.727
II	AMC, CTR, CIP, TE, TR, VA, CL, E	1	8	0.727
III	AMC, CXM, CTR, TR, VA, CL, E	1	7	0.636
*Klebsiella oxytoca*				
I	AMC, CTR, GEN, TE, VA, CL, E	1	7	0.636
II	AMC, CTR, TE, TR, VA, CL, E	1	7	0.636
*Pseudomonas aeruginosa*				
I	AMC, CXM, CTR, TE, VA	3	5	0.455
II	CXM, CTR, TE, VA	8	4	0.364
*Proteus mirabilis*				
I	VA	3	1	0.091
*Enterobacter cloacae*				
I	AMC, CXM, CTR, CIP, TE, TR	1	6	0.545
II	AMC, CXM, CTR, VA	3	4	0.364
III	AMC, CXM, CTR	2	3	0.273
IV	CTR	1	1	0.091
*Enterobacter hormaechei*				
I	AMC, CXM, CTR, VA	1	4	0.364
*Enterobacter kobei*				
I	AMC, CXM, CTR, VA	1	4	0.364

MARI=Multiple antibiotic resistance index, AMC=Amoxicillin-clavulanic acid, CXM=Cefuroxime, CTR=Ceftriaxone, CIP=Ciprofloxacin, TE=Tetracycline, TR=Trimethoprim, VA=Vancomycin, CL=Clarithromycin, E=Erythromycin

Table-3 illustrates the capacity of *Enterobacteriaceae* strains isolated from various food products to form biofilms as assessed by CV staining after 24 h at 37°C. All the biofilms were tested at the liquid-air interface. Overall, 83.33% of all tested *Enterobacteriaceae* isolates produced strong biofilms, while only 16.67% of two *K. pneumoniae* and three *E. cloacae* isolates generated moderate biofilms.

**Table-3:** Biofilm formation patterns of *Enterobacteriaceae* isolated from different food samples.

Bacterial isolates	Biofilm formation pattern			

	Biofilm former			Non-biofilm former (%)

	Strong (%)	Moderate (%)	Weak (%)	
*Pseudomonas aeruginosa* (n^¥^ = 11)	11 (100)	—	—	—
*Klebsiella pneumoniae* (n = 5)	3 (60)	2 (40)	—	—
*Klebsiella oxytoca* (n = 2)	2 (100)	—	—	—
*Proteus mirabilis* (n = 3)	3 (100)	—	—	—
*Enterobacter cloacae* (n = 7)	4 (57.14)	3 (42.86)	—	—
*Enterobacter hormaechei* (n = 1)	1 (100)	—	—	—
*Enterobacter kobei* (n = 1)	1 (100)	—	—	—
Ground total (n = 30)	25 (83.33)	5 (16.67)	—	—

^¥^n = Number of isolates

## Discussion

Foodborne zoonotic agents such as *Shigella*, *Salmonella*, and *E. coli* are the focus of most food safety research. This study focuses on emerging opportunistic *Enterobacteriaceae* (Ops) members, which are not well-known as foodborne pathogens but are still capable of causing infectious diseases. Thus, the current research aimed to determine the prevalence of various *Enterobacteriaceae* members in animal and poultry-derived foods such as beef, chicken meat, rabbit, milk, and eggs. The second goal was to characterize isolated members for virulence traits such as antibiotic resistance and biofilm formation capacity [26, 27]. Regarding prevalence data, the MALDI-TOF results identified four *Enterobacteriaceae* genera and seven species, including *K. pneumoniae*, *K. oxytoca*, and *P. aeruginosa*, *P. mirabilis*, *E. cloacae*, *E. hormaechei*, and *E. kobei*. *Pseudomonas aeruginosa* dominated the overall population of isolates from various food samples, accounting for 4.01% (11/274), followed by *E. cloacae* at 2.55% (7/274), and *K. pneumoniae* at 1.82% (5/274). Simultaneously, *P. mirabilis*, *K. oxytoca*, *E. hormaechei*, and *E. kobei* were confirmed in 1.09% (3/274), 0.73% (2/274), 0.36% (1/274), and 0.36% (1/274) of the tested samples, respectively. The majority of currently isolated *Enterobacteriaceae* are opportunistic and have been implicated in a clinical and public health crisis [25, 26]. To clarify, many hospital outbreaks were linked to *P. aeruginosa* contamination of medical equipment [28–30]. In addition, several *E. cloacae* outbreaks in hospitalized infants have been reported in recent years [31, 32]. *Klebsiella* *pneumoniae* gastrointestinal carriage, including foodborne, is a risk factor for liver abscess and has been linked to human sepsis [33]. The health significance associated with these isolates suggests that the food items investigated in this study could be potential sources and routes of illness spread. Previous research on retail beef and poultry with a similar goal to the current study revealed a high occurrence rate of *Enterobacteriaceae*, which in precisely calculated studies reached 95.2% and 100% of the total food sample. The most common genera and or species, however, differed, with *K. oxytoca*, *Serratia* spp., *E. coli*, and *Hafnia alvei* being the most common contaminants in the United States [11], and *Proteus* spp., *E. coli*, *Klebsiella* spp., and *Citrobacter* spp. being the most abundant in the Egyptian investigation [3]. Whilst the most abundant genera in Lagos, Nigeria were *Enterobacter* spp., *E. coli*, and *Klebsiella* spp. [34]. The *Enterobacteriaceae* family is a normal and healthy component of animal gut microbiota, which may explain its widespread distribution in tested animal and/or poultry foods. Furthermore, the origin of these bacteria, as well as multiple transmission routes during the production and handling of animal and/or poultry-derived foods [35, 36], prompted food safety authorities to adopt *Enterobacteriaceae* and/or their members as a valuable microbiological indicator of food safety, quality, and hygiene [20, 37, 38].

In terms of the detailed prevalence of *Enterobacteriaceae* species in relation to food category, *K. pneumoniae* was detected in 3.03% (2/66), 1.56% (1/64), 1.72% (1/58), and 3.70% (1/27) of tested chicken meat, rabbit, milk, and egg sample, respectively. Meanwhile, *K. oxytoca* was only identified in 3.13% (2/64) of rabbit meat. Similar *Klebsiella* isolation rates for chicken meat, rabbit, milk, and egg were previously reported by Gundogan and Avci [39], Gundogan *et al*. [40], Wu *et al*. [41], Huynh *et al*. [42], Jain and Yadav [43]. Although *K*. *pneumoniae* is a common cause of clinical and subclinical bovine mastitis in dairy cows [44, 45], its presence in the food chain may indicate unsanitary food preparation and handling procedures, undercooking, and poor storage conditions rather than causing human illness [46]. Besides, *P. aeruginosa* was isolated in 12.5% (8/64), 28.57% (2/7), and 1.72 (1/58) of rabbit, butter, and milk samples, respectively, which is consistent with previously published findings from the same food type [47–50]. Furthermore, *P. mirabilis* was encountered in 1.51 (1/66) and 3.45 (2/58) of chicken meat and milk samples, respectively, which was similar to previously reported rates in chicken meat [51, 52], but higher than other previously obtained levels [3, 53] from chicken meat and milk. Human UTIs, nosocomial infections, and wound infections have all been linked to *Proteus* [54, 55]. The main source of *Proteus* species transmission to humans within the food chain is poultry and derived products, which can occur through direct contact with live chickens or their fecal contaminated products [56–58]. In the current study, the incidence of *E. cloacae* in beef, chicken meat, rabbit, milk, and egg samples was 3.85% (2/52), 1.51% (1/66), 1.56% (1/64), 1.72% (1/58), and 3.70% (1/27), which is in accordance with the previous findings from comparable food types [43, 59–63]. To the best of our knowledge, the current study’s findings are the first to document the presence of three *Enterobacteriaceae* species in the tested foods. Two of these, *E. hormaechei* and *E. kobei*, were found in eggs at the same rate of 3.70%, and the third, *K. oxytoca*, was found in rabbit meat (1/27).

Focusing on the second objective of the current study, three distinct antimicrobial susceptibility patterns – non-MDR (non-MDR), MDR, and extensively multidrug-resistant (XDR) – were observed. Most of the *Enterobacteriaceae* isolates (n = 25) from this study had either MDR or XDR phenotypic profiles, with some isolates having a MARI of up to 0.727 and others having as few as 0.273. This is in contrast to five isolates, *P. mirabilis* (100%) and *E. cloacae* (40%), which had resistance to one antibiotic and a MARI of 0.091. Unfortunately, MDR pathogens, including species of *Enterobacteriaceae*, have recently been associated with nosocomial infections with high mortality rates. These MDR bacteria have the virulence to overcome the bactericidal effects of various antibiotic types or classes in both animals and humans [64, 65]. The majority of MDR microbes have emerged due to excessive antimicrobial drug use in animal feed. These bacteria are then exported into food supply through animal products such as milk, meat, and poultry [66, 67]. In this study, MDR *Enterobacteriaceae* were found to be highly prevalent, with 28 isolates having a MARI value >0.2 and each isolate being antibiotic-resistant to at least three different antibiotics. To specify, all currently isolated strains of *P. aeruginosa*, *E. hormaechei*, and *E. kobei* were demonstrated to be 100% MDR. In contrast, only 60% and 20% of *K. pneumoniae* and *E. cloacae* isolates were MDR, respectively. In addition, XDR patterns were identified in 100% of the isolates of *K*. *oxytoca* and *K*. *pneumoniae*. These MDR *Enterobacteriaceae* isolates, also referred to as superbugs, are capable of horizontally transferring their resistance genes to other pathogenic microorganisms at various points along the food chain and have few effective treatments for their infections [17, 67]. The increasing role of animal-derived food to MDR *Enterobacteriaceae*, including *Enterobacter*, *Klebsiella*, and *Serratia*, have previously been documented in raw milk, red meat, and chicken meat this trend continues [17, 34, 68–70]. Of these health significances**,**

according to a Centers for Disease Control and Prevention report from 2013, fluoroquinolone-resistant *P*. *aeruginosa* and other antibiotic-resistant infections sickened more than two million people each year, with at least 23,000 dying [71]. Furthermore, data from the European AMR Surveillance Network revealed that high levels of antibiotic resistance persisted in the European Union between 2014 and 2017 for several bacterial species, with *K*. *pneumoniae* being among the most resistant to at least one of the antibiotic groups of aminoglycosides, cephalosporins, fluoroquinolones, and carbapenems [72]. Nonetheless, it should be noted that some of the currently observed phenotypic resistance could be intrinsic in some members, such as TE resistance in *P*. *aeruginosa* [73, 74], carbapenem resistance in *K*. *pneumoniae* [75], and beta-lactam resistance in *E*. *cloacae* [76]. However, current phenotypic resistance findings confirm the rising failure of critical antimicrobials to control infections caused by MDR isolates. Among these findings, it was found that all isolated strains, except *K*. *oxytoca* and *P. mirabilis*, were resistant to critically important beta-lactamase inhibitor antibiotics. Resistance to these antibiotics, which include AMC, second-generation cephalosporins (CTR), and 3GCs (CXM), have undesirable consequences because they are approved to treat serious infections such as Salmonellosis, pneumonia, intra-abdominal infection, sepsis, and febrile neutropenia. Furthermore, instead of fluoroquinolones, which interfere with cartilage formation, this class of beta-lactamase inhibitor antibiotics is prescribed to treat serious infections in children and pregnant women [77, 78]. Before this study, it was determined that 71.4% of *Enterobacter* spp. and 100% of *Klebsiella* spp. isolated from sausage were resistant to CTX and AMC, respectively [79]. Furthermore, combined resistance to these antimicrobial classes, particularly 3GCs, has been attributed primarily to extended-spectrum beta-lactamase (ESBL), which is mostly plasmid-borne [75]. The ESBL comprises resistance determinants for other antimicrobial classes, such as carbapenems, which are used as last-line antibiotics and may be used as alternatives in treating patients infected with serious MDR Gram-positive and GNB [75, 80]. The most serious threat was the emergence of *Enterobacteriaceae* species, such as *K. pneumoniae*, which contain intrinsic carbapenemases (KPCs), enzymes that can adversely affect the efficacy of carbapenems, and other members, such as *P*. *aeruginosa*, that can acquire such mobile resistance genetic elements [75]. Similarly, members of the *E. cloacae* complex showed a special capacity to acquire genes encoding resistance to various classes of antibiotics, such as a number of KPC genes, in addition to intrinsic beta-lactam resistance [76]. Such *E. cloacae* has been classified as a “priority pathogen” due to its clinical importance [81, 82]. Current phenotypic resistance results of all isolated *K*. *pneumoniae*, *P. aeruginosa*, *E. cloacae*, *E. hormaechei* and *E. kobei* to different antibiotic classes, including diaminopyrimidines, fluoroquinolones, glycopeptides, lincosamide, macrolide, and TEs, support earlier findings that beta-lactam resistant *Enterobacteriaceae* species could carry resistance determinants for other antibiotics through a variety of mechanisms, including sequential chromosomal mutations of the overproduction of intrinsic beta-lactamases, hyper-expression of efflux pumps, target modifications, and permeability alterations [75]. Resistance to beta-lactam antibiotics, including cephalosporins (CXM, CTR), has previously been documented in *P. aeruginosa*, *K*. *pneumoniae*, and *E. cloacae* [59, 83–85]. *Proteus mirabilis* is naturally resistant to several antibiotics, including colistin, and has a lower susceptibility to imipenem, as well as the ability to acquire several antibiotic resistance genes, including beta-lactamases genes [86], as documented in a previous case in chicken [51]. In this study, isolated *P. mirabilis* was completely susceptible to all 11 antibiotics tested, including beta-lactam antibiotics, but completely resistant to VA (n = 3). The lack of resistance in *P. mirabilis* in this study could imply that antibiotics are not commonly used in poultry and animal management in the studied area.

Improper antimicrobial agent application, such as incorrect indication, duration, and route of administration in humans, and growth promotion, and prophylaxis in animals [87], as well as negative thought patterns of using leftover antibiotics from a family member, and improper antibiotic cessation, may be linked to the development of MDR pattern bacteria [88–90]. The high prevalence of MDR and XDR *Enterobacteriaceae* isolated from food under investigation necessitates increased efforts from the medical and veterinary sectors to rationalize and impose more restrictions on the accessibility and application of antibiotics. In addition, it places additional pressure on the Egyptian National Food Safety Authority to implement stringent monitoring programs for animal-derived food to prevent the spread of these potentially harmful threats.

Concerning the other threat of biofilm formation, 83.33% of the currently isolated *Enterobacteriaceae* from various food products exhibited strong biofilm production traits, while only 16.67% exhibited moderate biofilm production. At the species level, current isolates of *P. aeruginosa*, *P. mirabilis*, *K. oxytoca*, *E. hormaechei*, and *E. kobei* demonstrated robust biofilm formation. While *K. pneumoniae* and *E. cloacae* formed strong biofilms in 60% and 57.14% of cases, respectively, and moderate biofilms in 40% and 42.86% of cases, respectively. Numerous previous studies have been conducted in light of this threat. Some have documented a strong capacity for biofilm formation in *Enterobacteriaceae* isolated from various animal-derived foods such as chicken and meat [3, 91], goat milk (90% of isolates) [92], as well as *K*. *pneumoniae* and *Enterobacter* spp, isolated from stainless steel pipe surfaces in milk processing plants [93]. In addition, a previous study has shown that 100% *K*. *pneumoniae* and 84% *K*. *oxytoca* had a strong biofilm capacity after 24 h of incubation [94]. Biofilm is one of the most influential indicators of bacterial pathogenicity. In addition, for various reasons, it has a negative impact on a number of food processing steps, such as storage, processing, and preservation, with negative consequences for both the economy and human health. To summarize these effects, biofilms are a major cause of food spoilage, outbreaks, and damage to food processing machinery [30]. Biofilm protects bacteria from antibiotics and phagocytosis in the medical field. Biofilm promotes bacterial survival in the food industry while also creating an environment conducive to the exchange of antibiotic resistance genes [3], which then plays a significant role in AMR. Furthermore, biofilms can contain pathogenic bacteria and spoilage, increasing post-processing contamination when they detach or slough off into foods and posing a risk of foodborne infection to the general public [95, 96]. Biofilm formation makes bacteria resistant to sanitizers and disinfectants, requiring the food industry to change its cleaning and disinfection dynamics expensively [30].

## Conclusion

The MALDI-TOF results identified seven *Enterobacteriaceae* species, with *P. aeruginosa* dominating the overall population of isolates from various food samples, followed by *E. cloacae* and *K. pneumoniae*. To the best of our knowledge, the current study’s findings are the first to confirm the presence of three *Enterobacteriaceae* species in eggs, including *E. hormaechei* and *E. kobei*, with a third, *K. oxytoca*, found in rabbit meat. The majority of the 25 *Enterobacteriaceae* isolates from the current study had either MDR or XDR phenotypic profiles, with some isolates having a MARI of up to 0.727 and others having as few as 0.273. Current phenotypic resistance findings suggest the increasing failure of critical antimicrobials to control infections caused by MDR isolates. This analysis revealed that, except two isolated strains, all were resistant to the crucial beta-lactamase inhibitor antibiotics. According to current phenotypic resistance results of the majority of isolates to various tested antibiotic classes, beta-lactam resistant *Enterobacteriaceae* species shared phenotypic resistance for other critically important antibiotics. While 83.33% of the currently isolated *Enterobacteriaceae* from various food products exhibited strong biofilm production traits, only 16.67% exhibited moderate ability. Foodborne *Enterobacteriaceae* with MDR and strong biofilm indices threaten several food processing steps, such as storage, processing, and preservation, which could have negative economic and public health effects. The findings of this study could provide Egyptian food safety authorities with the data they need to determine the actual implications of enterobacteria in animal-derived food, as well as recommend public health officials implement more effective strategies to reduce contamination rates and mitigate the impact of these bacteria on public health.

## Data Availability

The supplementary data can be available from the corresponding author upon a reasonable request.

## Authors’ Contributions

SNE: Conceptualization, methodology, data curation, resources, funding acquisition, project administration, writing – review and editing. AH: Conceptualization, methodology, formal analysis, visualization, writing – review and editing. DABA: Formal analysis, methodology, roles/writing-original draft. IIS: Investigation, supervision, validation, writing – review, and editing. All authors have read, reviewed, and approved the final manuscript.
